# Buforin IIb induced cell cycle arrest in liver cancer

**DOI:** 10.1080/19768354.2019.1595139

**Published:** 2019-04-26

**Authors:** Dan Li, Yong Xu

**Affiliations:** a Department of Geriatrics, The Second Xiangya Hospital of Central South University, Changsha, People’s Republic of China; b Blood Purification Center, The Third Xiangya Hospital of Central South University, Changsha, People’s Republic of China

**Keywords:** Buforin IIb, liver cancer, apoptosis, cell cycle

## Abstract

The inhibitory effect of buforin IIb on different types of cancer, although not liver cancer, has been demonstrated previously. The aim of the present study was to investigate the effects of buforin IIb on the progression of liver cancer. The human liver cancer cell line HepG2 was treated with purified buforin IIb and the cell activity was determined by MTT, colony formation and transwell assays. The protein expression levels of cyclin-dependent kinases (CDKs) and cyclins were analyzed by western blotting and immunofluorescent cell staining. A tumor growth model was constructed using nude mice, and buforin IIb treatment was administered. The levels of CDK2 and cyclin A in the tumor tissues were detected by western blotting. Buforin IIb treatment depressed cell viability and colony formation and induced apoptosis significantly, and 1.0 µM concentration of buforin IIb was found to be the optimal dosage. The cell cycle was arrested at the G2/M phase following buforin IIb treatment. CDK2 and cyclin A were downregulated by treatment of the cells with 1.0 µM buforin IIb for 24 h. Treatment with buforin IIb also inhibited the migration of liver cancer cells in vitro. Furthermore, 50 nmol buforin IIb injection suppressed HepG2 cell subcutaneous tumor growth in the nude mouse model. Similar to the in vitro results, buforin IIb injection reduced the expression of CDK2 and cyclin A in the tumor tissue. these results demonstrate that buforin IIb inhibited liver cancer cell growth via the regulation of CDK2 and cyclin A expression.

## Introduction

Metastatic liver tumors represent the most prevalent cancers in adults (Bosch et al. 2004). As a leading cause of morbidity and mortality worldwide, liver cancer affects survival and the quality of life (Bosch et al. 2004). Currently, resection surgery, radiation therapy and chemotherapy are the standard treatments used for patients with liver cancer (Baskar et al. 2012). However, severe side effects arise following radiation therapy and chemotherapy (Lawrence et al. 1995; Wulf et al. 2006). Furthermore, these treatments have shown limited curative effects and poor prognosis in clinical practice (Bruix and Sherman 2011). The development of novel agents that specifically target liver cancer is, therefore, urgently required to improve the treatment of liver cancer.

Several cationic antimicrobial peptides have been reported to display anticancer activity (Hancock 2001). Buforin II is an antimicrobial peptide derived from histone H2A with a helix-hinge-helix structure, which was first isolated from the stomach tissue of the Asian toad (Bufo bufo gargarizans) (Park et al. 1996). The peptide contains 21 amino acids and exhibits a strong antimicrobial activity against a variety of microorganisms, including bacteria and fungi without hemolytic activity (Park et al. 1998). Due to its unique structure, buforin II is able to rapidly cross the membranes of bacteria without lysing cells and kills the bacteria by the destruction of intracellular macromolecules (Park et al. 2000). An analog of buforin II, known as buforin IIb has been developed, which contains an α-helical sequence at the end of the C-terminus and has a stronger cytolytic activity than buforin II in microorganisms (Jang et al. 2011; Jang et al. 2012). Buforin IIb exhibits antitumor activities by specifically targeting cancer cells via interaction with cell surface gangliosides (Lee et al. 2008). In a study conducted by Lee et al., the effects of buforin IIb on cancer cell lines, including leukemia, central nervous system tumor, non-small cell lung cancer, melanoma and renal cancer cell lines were demonstrated (12). The authors also used a NCI-H460 lung cancer cell line transplant to form tumor xenografts and demonstrated that buforin IIb treatment was able to suppress cancer development *in vivo* (Lee et al. 2008). These findings indicate that buforin IIb is a potential novel therapeutic agent for the treatment of cancers.

Although antitumor effects have been illustrated in leukemia, central nervous system tumors, non-small cell lung cancer, melanoma and renal cancer, the potential curative effect of buforin IIb on liver cancer has not yet been unveiled. Therefore, the present study investigated the anticancer activity of buforin IIb in liver cancer and analyzed the mechanism of cancer cell-killing in vitro and *in vivo*. The purpose of this study was to explore the curative value of buforin IIb in the treatment of liver cancer.

## Materials and methods

### Peptides

Buforin IIb [amino acid sequence, RAGLQFPVG(RLLR)3] was supplied in crude form by GL Biochem Ltd. (Shanghai, China). The crude peptide was then purified to near homogeneity by reversed phase high-performance liquid chromatography using a 0.46 × 25-cm Vydac 214TP54 C4 column equilibrated with acetonitrile, water and trifluoroacetic acid (35.0:64.9:0.1, v/v/v) at a flow rate of 6 ml/min. The acetonitrile concentration was increased to 65% (v/v) after 60 min using a linear gradient. The purity of the peptides was N98%, as determined by electrospray mass spectrometry. The peptide was highly soluble in physiological buffers.

### Cell lines and animal models

The human liver cancer cell line HepG2 was purchased from the Shanghai Cell Bank at the Chinese Academy of Sciences (Shanghai, China). The culture conditions for the cells were a temperature of 37°C in a 5% CO2:95% air-humidified atmosphere in Dulbecco’s modified Eagle’s medium supplemented with 10% fetal bovine serum and 2 mM L-glutamine. The cell passage was performed using 0.5% trypsin-ethylenediamine tetraacetic acid. Five-week-old female BALB/c nude mice were purchased from the Chinese Academy of Medical Sciences. Experiments were approved by the ethics committee of the Second Xiangya Hospital of Central South University (Changsha, China).

### 3-(4,5-Dimethylthiazol-2-yl)-2,5-diphenyltetrazolium bromide (MTT) assay

The cells were first seeded into 96-well plates at a density of 1 × 103 cells/well. After 24 h culture, the cells were treated using buforin IIb for different times (0, 4, 8, 12, 24 and 48 h treatment at 1.0 µM concentration; *n* = 3 for each group) or different dosages (24 h treatment at 0.1, 0.5, 1.0, 5.0 and 10.0 µM concentration; *n* = 3 for each group). Cells incubated with dimethylsulfoxide (DMSO) were used as control. After treatment, the cells were stained using 0.5 mg/ml MTT solution (Sigma-Aldrich, St. Louis, MO, USA) for 4 h. The medium was then replaced with 500 ml DMSO and the optical density (OD) was measured with a microplate reader (Bio-Rad Laboratories, Inc., Hercules, CA, USA). The cell viability was calculated using the formula: % viability = (OD treated sample/OD untreated sample) × 100 using the OD570 value.

### Colony formation assay

For each well, 2 × 10^3^ cells/well were seeded into 6-well plates and incubated for 7 days (*n* = 3 for each group). After washing three times using phosphate-buffered saline (PBS), the cells were fixed in methanol and stained with crystal violet for 10 min. The colonies were detected using an IX71 inverted microscope (Olympus Corporation, Tokyo, Japan).

### Cell cycle analysis

Cells were treated with buforin IIb for different times or different dosages (*n* = 3 for each group) as described in the MTT assay. DMSO was used as the control. The cells were then stained using 0.05 mg/ml Propidium Iodide (PI) (Jiamay Biotech, Beijing, China). Briefly, the cells were first washed with 10 mM PBS and centrifuged at room temperature for 5 min. The sediment was resuspended in binding buffer and then incubated with PI for 30 min. Finally, the cells were analyzed using a Cytomics FC 500 MPL cytometer (Beckman Coulter, Inc., Miami, FL, USA).

### Western blotting

The protein expression of cyclin-dependent kinases (CDKs) and cyclins was analyzed using western blot assays. Primary rabbit polyclonal antibodies against CDKs and cyclins were purchased from Sigma-Aldrich. The secondary antibody was horseradish peroxidase (HRP)-conjugated anti-rabbit IgG (Sigma-Aldrich). After homogenization using radioimmunoprecipitation assay buffer (Beyotime Institute of Biotechnology, Wuhan, China), the proteins from tissues or cells were extracted and quantified using a BCA protein assay kit (Beyotime Institute of Biotechnology). For each sample, 10 µg total proteins were electrophoresed on 15% sodium dodecyl sulfate-polyacrylamide gel and transferred to a poly-vinylidene difluoride membrane (EMD Millipore, Billerica, MA, USA). The membrane was then blocked using 4% skimmed milk for 1 h at room temperature and incubated with primary antibodies against CDK1 (1:1000), CDK2 (1:1000), CDK4 (1:800), cyclin (1:500), cyclin B (1:1000) and glyceraldehyde 3-phosphate dehydrogenase (GAPDH; 1:2000) at 4°C overnight. After washing with Tris-buffered saline and 84 Tween 20 (TBST) buffer (pH 7.6, 20 mM Tris-HCl, 137 mM NaCl, 0.01% Tween-20), the membranes were incubated with secondary antibody and visualized using enhanced chemiluminescence reagents (ECL; EMD Millipore).

### Immunofluorescent cell staining

HepG2 cells were seeded onto coverslips coated with poly-L-lysine at a density of 5 × 10^4^ per coverslip. After 24 h culture, the cells were washed with PBS and treated with 1.0 µM buforin IIb for 24 h at 37°C. The cells were then washed with PBS to clear the buforin IIb, and fixed with 4% paraformaldehyde. After incubation with primary antibodies against CDK2 (1:200) and cyclin A (1:100) at room temperature for 2 h, the cells were co-stained using 4’,6-diamidino-2-phenylindole (DAPI) to stain the cell nuclei. The signals were observed under a laser scanning confocal microscope (Olympus Fluoview FV1000; Olympus Corporation).

### *In vitro* migration assay

Cell migration was detected using Transwell analysis (24-well insert; pore size, 8 mm; BD Biosciences, Franklin Lakes, NJ, USA; *n* = 3 for each group). The cells were first treated with 1.0 µM buforin IIb for 24 h and then plated (5 × 10^4^) into the upper chambers in serum-free medium. The lower chambers were filled with serum-containing medium. After 24 h culture, the cells were stained using crystal violet and observed using an IX71 inverted microscope.

### Tumor growth in a nude mouse model

A HepG2 cell subcutaneous tumor model was established in mice as described below and used to investigate the effect of buforin IIb on tumor growth (*n* = 3 for each group). The tumor formation model was generated by the injection of HepG2 cells (2 × 10^5^ cells in 0.1 ml PBS) subcutaneously into the right shoulder of the mouse. After the tumors reached 50 mm^3^ in volume, buforin IIb (50 nmol in 100 ml PBS) or injection buffer (DMSO in 100 ml PBS) was administered by injection into the tail vein every 2 days. Tumor volumes were calculated every 3 days using the following formula: Volume = length × width2 × 0.52. After weeks, mice were sacrificed and tumor tissues were assayed by western blot analysis and immunofluorescent cell staining.

### Terminal deoxynucleotidyl-transferase-mediated dUTP nick end labeling (TUNEL) staining assay

To detect cell apoptosis in the tumor tissues, tissue sections were prepared as described above. TUNEL staining was performed according to the manufacturer's protocol (Roche Diagnostics, Indianapolis, IN, USA). The nuclei were stained using DAPI. Signals of TUNEL-stained cells were detected using a laser scanning confocal microscope (Olympus Fluoview FV1000).

### Statistical analysis

All experiments were repeated three times. Data are expressed as mean ± standard error of the mean (SEM). Differences among groups were analyzed by one-way analysis of variance conducted using the SPSS version 17 software package (SPSS, Inc., Chicago, IL, USA). *P* < 0.05 was considered to indicate a statistically significant difference.

## Results

### Buforin IIb depresses the tumorigenicity of liver cancer cells

**Figure 1.**
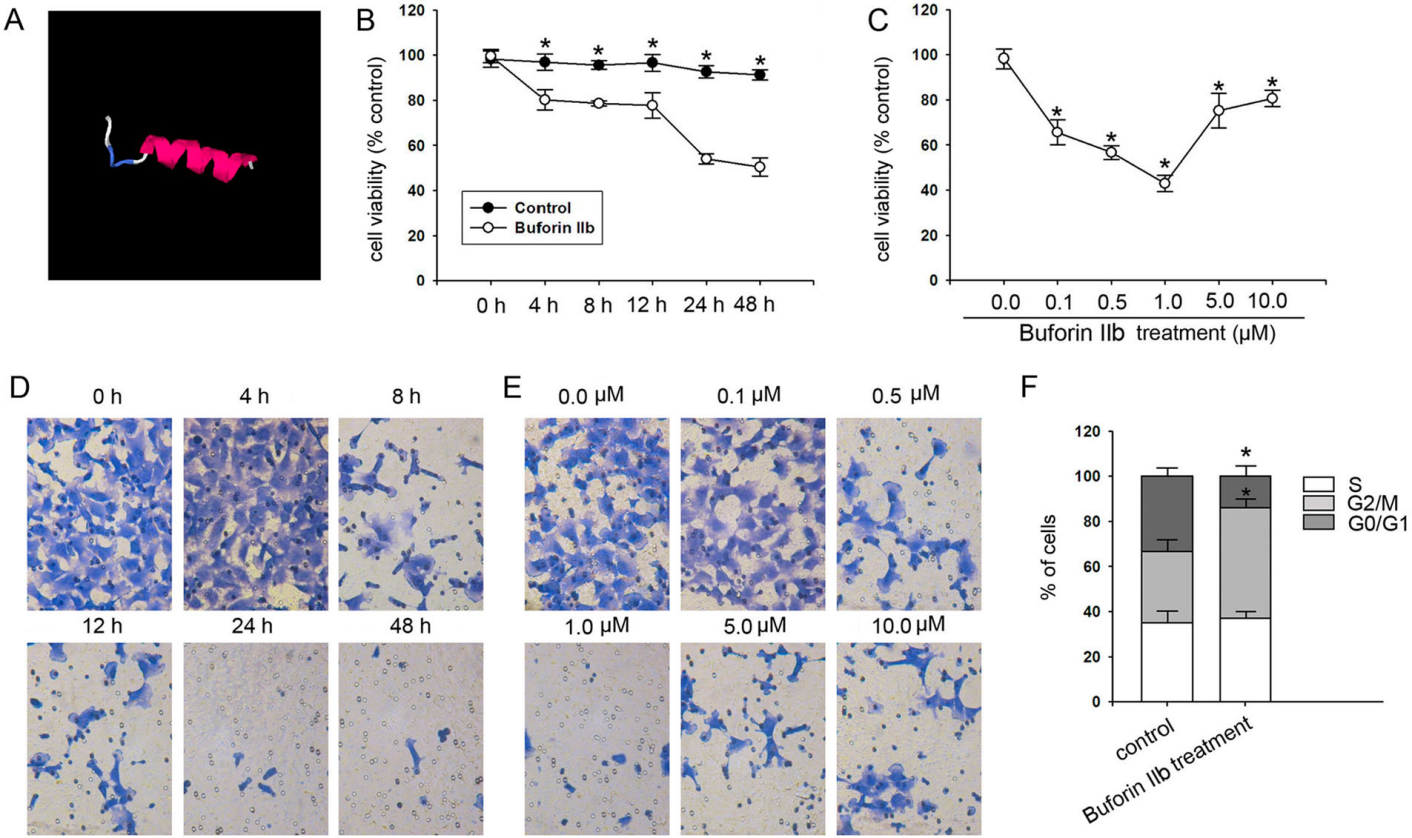
*In vitro* antitumor effect of buforin IIb on liver cancer cells. (A) Structural model of buforin IIb predicted and generated by the I-TASSER server. Effect of buforin IIb on cell viability at various (B) times and (C) concentrations (*n* = 3). *P < 0.05 vs. the control. Effect of buforin IIb on colony formation at varying (D) times and (E) concentrations. (F) Phases of the cell cycle following treatment with buforin IIb (*n* = 3). *P < 0.05 vs. the control.

The peptide buforin IIb has been demonstrated to have antitumor effects on several types of cancer, although not on liver cancer. In order to investigate the potential of buforin IIb as a drug for liver cancer, buforin IIb was obtained and purified. A structural representation of this peptide is shown in Figure 1(A). The anticancer activity of buforin IIb was evaluated in HepG2 liver cancer cells. Following 4, 8, 12, 24 and 48 h treatment with 1.0 µM buforin IIb, cell viability was decreased significantly and in a time-dependent manner (*P* < 0.05; Figure 1(B)). The 24-h treatment with buforin IIb at 0.1, 0.5, 1.0, 5.0 and 10.0 µM concentrations also significantly decreased the cell viability (Figure 1(C)). However, no dosage-dependent effect was observed. The 1.0 µM concentration of buforin IIb exhibited the optimal depressive effects on cell viability. Similarly, treatment with buforin IIb after 4, 8, 12, 24 and 48 h suppressed colony formation in liver cancer cells and 24 h of treatment with buforin IIb exhibited the greatest inhibitory effect on colony formation (Figure 1(D)). Buforin IIb at 0.1, 0.5, 1.0, 5.0 and 10.0 µM also inhibited colony formation. Therefore, 1.0 µM concentration of buforin IIb had the greatest inhibitory effect on colony formation (Figure 1(E)). Next, Flow cytometry was used to detect the apoptosis and cell cycle distribution following buforin IIb treatment. Considering 1.0 µM was the optimal dosage of buforin IIb, the cells were assayed after treatment with 1.0 µM buforin IIb for 24 h. Cell cycle analysis indicated that the cells were significantly arrested at the G2/M phase following buforin IIb treatment compared with the control (Figure 1(F)). These results suggested that buforin IIb inhibits cell proliferation and promote apoptosis in liver cancer.

### Buforin IIb regulates CDK2 and cyclin A expression.

**Figure 2.**
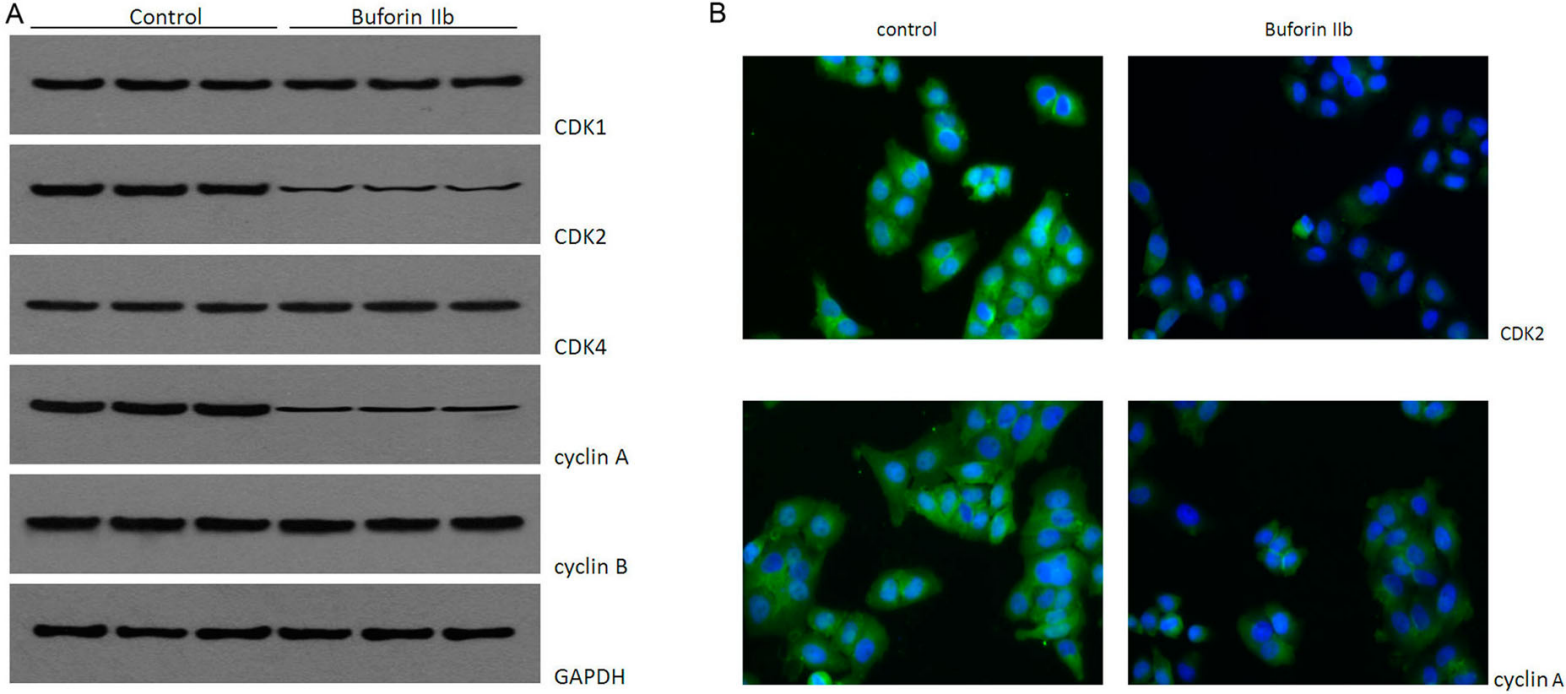
Buforin IIb reduced the expression of CDK2 and cyclin A in liver cancer cells. (A) Western blot analysis showing the expression of CDK1, CDK2, CDK4, cyclin A and cyclin B after buforin IIb treatment. (B) Immunofluorescent cell staining confirmed the inhibitory effect of buforin IIb on CDK2 and cyclin A.

Previous study showed Buforin IIb has an inhibitory effect on cell cycle**.** The cell cycle is controlled by CDKs and cyclins, including CDK1, CDK2, cyclin A, cyclin B and cyclin D. Thus, to understand the molecular mechanism by which buforin IIb arrests cells at the G2/M phase, the expression levels of these proteins were analyzed following buforin IIb treatment. A 1.0 µM concentration of buforin IIb was used to treat the HepG2 cells for 24 h. The results of western blot analysis showed that, with the exception of CDK2 and cyclin A, these proteins underwent no significant changes after buforin IIb treatment. However, CDK2 and cyclin A levels decreased notably following treatment with 1.0 µM buforin IIb for 24 h (Figure 2(A)). Similarly, the immunofluorescent cell staining showed that after treatment, the protein levels of CDK2 and cyclin A were suppressed by buforin IIb (Figure 2(B)). Therefore, buforin IIb suppress cell cycle by regulation CDK2 and cyclin A expression in the liver cancer.

### Buforin IIb inhibits liver tumor cell migration *in vitro*

**Figure 3:**
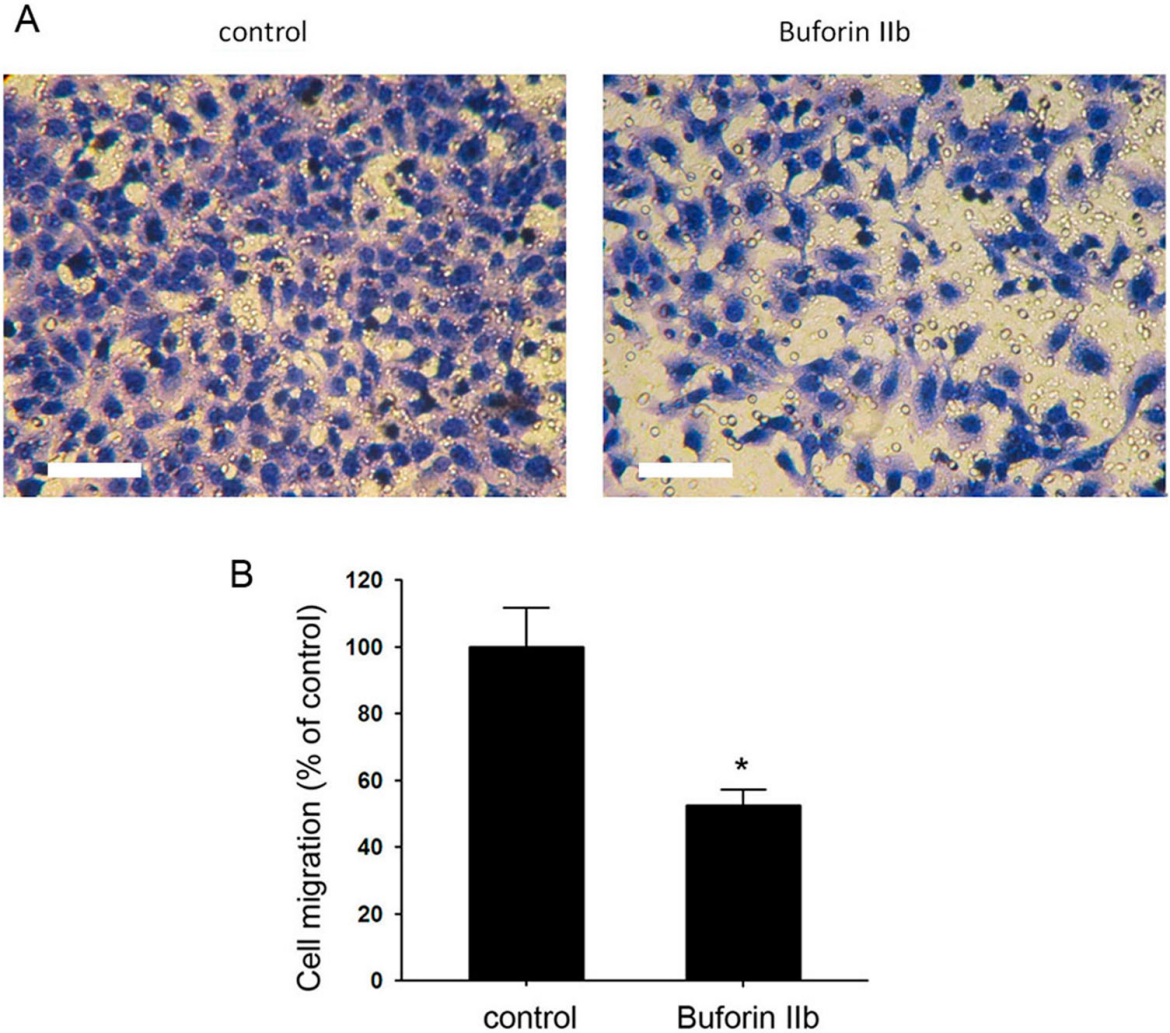
Buforin IIb inhibited the migration of liver cancer cells in vitro. HepG2 cells were treated with 1.0 μM buforin IIb for 24 h. (A) Representative images of migrated liver cancer cells. Scale bar = 50 μM. (B) Quantification of the percentage of the migrated cells. n = 3. *P < 0.05 vs. the control.

The generation of metastatic tumors is dependent upon the migration of tumor cells. The effect of buforin IIb on cell migration was thus investigated. HepG2 cells were treated with 1.0 µM buforin IIb for 24 h and then the ability of the cells to migrate was detected using a Transwell assay system. The results indicated that at a concentration of 1.0 µM, buforin IIb inhibited cell migration significantly compared with that of the control group (Figure 3(A)). Cell migration was decreased by 47.62% in the buforin IIb group compared with that in the control group (Figure 3(B)). Our results suggest an inhibitory role of buforin IIb in cell migration.

### Buforin IIb inhibits tumor growth *in vivo*

**Figure 4.**
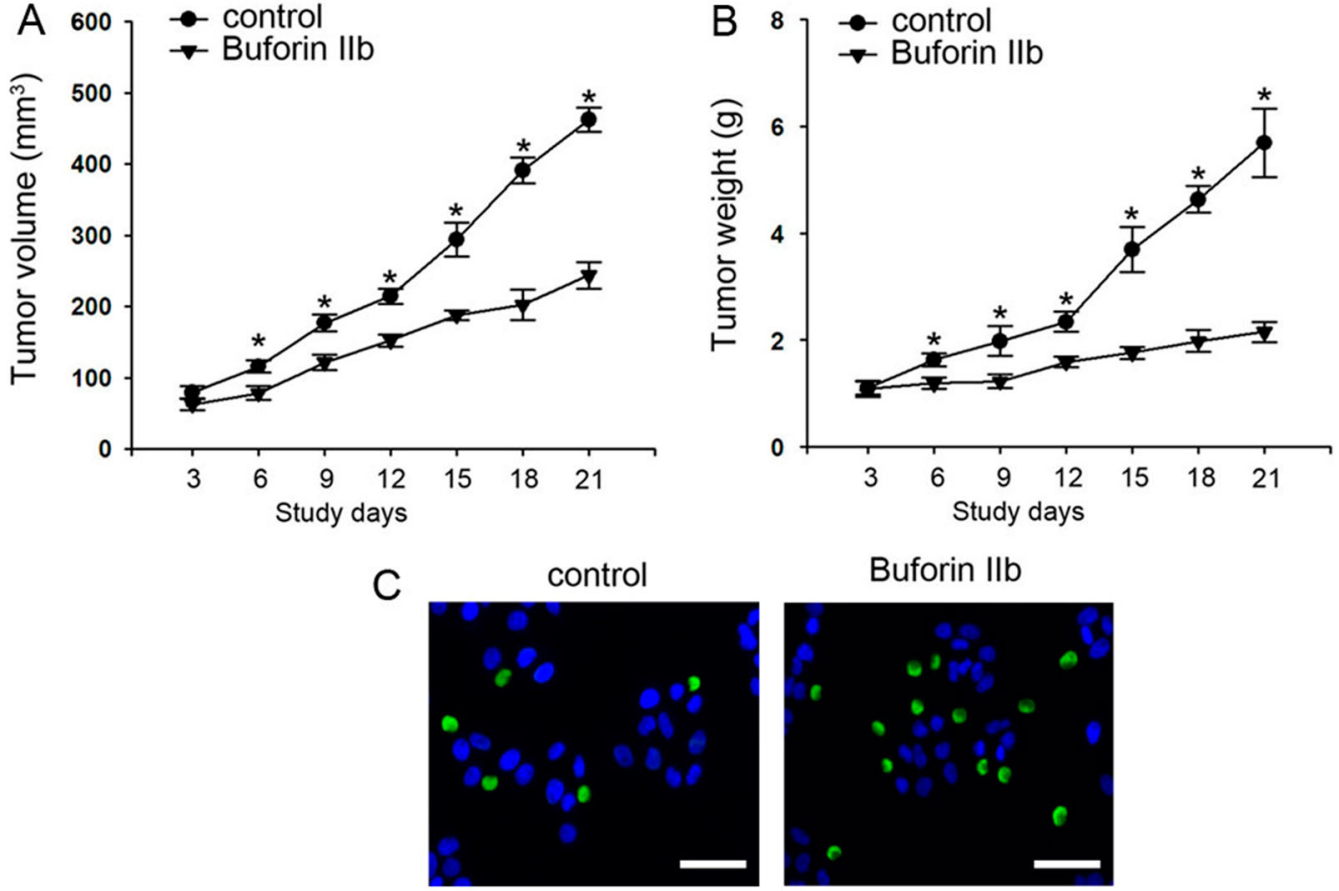
Tumor formation and TUNEL assay of the tumor tissues. Changes in (A) tumor volume and (B) tumor weight after treatment with 50 nmol buforin IIb, (n = 3). *P < 0.05 vs. the control. (C) TUNEL assay results showed that 50 nmol buforin IIb promoted apoptosis in tumor tissues. TUNEL, terminal deoxynucleotidyl-transferase-mediated dUTP nick end labeling.

Following determination of the effect of Buforin IIb on liver tumor cells in vitro, whether this peptide could inhibit tumor growth *in vivo* was next determined. In order to investigate the effect of Buforin IIb in tumor growth of HepG2 xenografts, 6-week-old male mice were inoculated with HepG2. After the tumor volume reached by 50 mm^3^, 50 nmol buforin IIb or injection buffer (DMSO in 100 ml PBS) as control were injected by tail vein injection every 2 days. The tumor volume and tumor weight were significantly inhibited in the buforin IIb peptide-treated mice compared with control from days 6 to 21 of treatment (*P* < 0.05; Figure 4(A,B)), which is consistent with the in vitro findings. Furthermore, the TUNEL assay results showed that buforin IIb treatment induced cell apoptosis in the tumor tissue (Figure 4(C)).

### Evaluation of CDK2 and cyclin A expression following buforin IIb treatment *in vivo*

**Figure 5.**
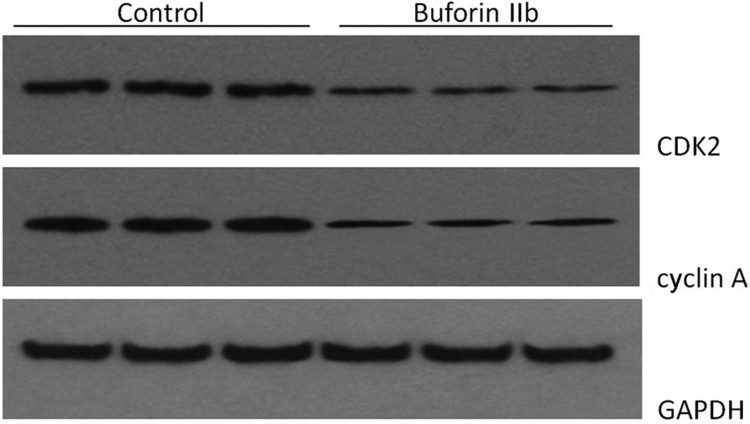
Buforin IIb repressed the expression of CDK2 and cyclin A in tumor tissues *in vivo*.

The effects of buforin IIb on CDK2 and cyclin A in xenograft tumor tissues were analyzed by western blotting and immunohistochemistry. The western blotting results were consistent with the results of the in vitro experiments; buforin IIb treatment repressed the expression of CDK2 and cyclin A in xenograft tumor tissues (Figure 5).

## Discussion

The present study first determined the primary finding that buforin IIb suppressed the progression of liver cancer. Treatment with buforin IIb inhibited the expression of CDK2 and cyclin A in HepG2 cells, which contributed to disruption of the cell cycle and arrested the cell cycle at the G2/M phase. Furthermore, buforin IIb also suppressed cell migration in vitro. The results of the *in vivo* experiments showed that buforin IIb inhibited tumor growth, and similar to the in vitro findings, the expression levels of CDK2 and cyclin A in the tumor tissue were inhibited by buforin IIb treatment.

Previous studies have identified several peptides that are able to significantly depress tumor progression and could 17 be potential antitumor therapies(Cao and Lin 2006; Hoskin and Ramamoorthy 2008). However, until now, all the investigated peptides have shown either limited effects on tumors or non-specificity for tumor cells, which leads to injury of normal cells. The emergence of buforin II, however, provides a promising therapeutic strategy. In addition to exhibiting strong antitumor effects, buforin II has also been revealed to be highly specific against tumor cells in comparison with normal cells (Park et al. 1998; Park et al. 2000). On the basis of buforin II, Lee et al. developed a new buforin II analog, buforin IIb, which had a stronger cytolytic activity against cancer cells than does buforin II (Lee et al. 2008). The authors demonstrated cytolytic activity for buforin IIb against several types of tumor cells but not liver cancer. In the present study, it was demonstrated that buforin IIb is able to depress tumor proliferation and cell migration.

According to previous studies, buforin IIb is able to exert effects on several types of cancer cells, with remarkable selectivity for cancer cells, and it has been indicated that the anticancer action of buforin IIb involves the induction of cancer cell apoptosis (Jang et al. 2011; Jang et al. 2012; Lee et al. 2008; Wang et al. 2011). However, the mechanism of the apoptosis induced by buforin IIb remains unclear. The present study demonstrated that buforin IIb is a potential anti-cancer drug that can accelerate the apoptosis of liver cancer cells as a result of arresting the cell cycle at the G2/M phase. The molecular mechanism of aberrant cell cycles may be associated with the abnormal expression of cell-cycle genes, such as CDKs and cyclins.

The findings of the current study are consistent with this; the expression levels of CDK2 and cyclin A were significantly decreased following buforin IIb treatment, which may affect the cell cycle and arrest the cell cycle at the G2/M phase. CDK2 and cyclin A are key factors in cell cycles, and act by controlling the pathway of DNA synthesis (Jackman and Pines 1997; Malumbres and Barbacid 2009; Yin et al. 2001). Oncogenic disruption of the cell cycle machinery (such as upregulation of CDK2 and cyclin A) is a universal phenomenon in liver cancer (Lu et al. 1997; Molenaar et al. 2009; Shapiro 2006). In addition, previous studies have shown that the lack of these two genes induces cell apoptosis (Kasten and Giordano 1998; Meikrantz et al. 1994; Rivera et al. 2006). The present study is the first to demonstrate the inhibitory effects of buforin IIb on CDK2 and cyclin A, which unveils the molecular basis by which buforin IIb affects the cell progression of liver cancer cells.

The results of the present study demonstrated that buforin IIb inhibits the progression of liver cancer both in vitro and *in vivo*. These findings indicate that buforin IIb has potential as a therapeutic drug for the treatment of liver cancer. In the present study, buforin IIb inhibited the viability and colony formation of liver cancer cells and tumor formation in a nude mouse model. Furthermore, the inhibitory effects of buforin IIb on CDK2 and cyclin A were shown. The decreased expression of CDK2 and cyclin A is likely to have disturbed the cell cycle and arrested the cells at the G2/M phase. Since buforin IIb has been demonstrated to have antitumor effects in leukemia, central nervous system tumors, non-small cell lung cancer, melanoma and renal cancer (Lee et al. 2008), the inhibition of liver cancer by buforin IIb may have been expected. However, the present study is the first to demonstrate that buforin IIb inhibits cell proliferation by regulating the cell cycle via mediating the expression of CDK2 and cyclin A. This information extends the scope of the understanding of buforin IIb's effects on cancer progression.

In conclusion, the present study demonstrates that buforin IIb suppresses the progression of liver cancer by inducing cell apoptosis, and inhibiting cell viability, colony formation and cell migration. Furthermore, it found that buforin IIb treatment depresses CDK2 and cyclin A expression. *In vivo* experiments using a nude mouse model suggested that buforin IIb inhibits tumor formation. These results indicate that buforin IIb could be a potent therapeutic drug for liver cancer.
